# Transfusion-associated malaria.

**DOI:** 10.3201/eid0202.960216

**Published:** 1996

**Authors:** F Taylor


### Transfusion-Associated Malaria

**To the Editor:** A recent article by Zucker (1) described two cases of malaria that were probably transfusion associated. A case of transfusion-associated malaria in which the source *was* identified was reported in San Francisco in 1991. The case was in an elderly man in whom malaria infection developed after coronary bypass surgery.

The patient was born in China and immigrated to the United States in 1940. His only travel outside the United States was a trip to Hong Kong in 1951 for 6 months. The patient's wife was born in China and had malaria in 1941 during World War II. She received no treatment at that time or at any other time. She came to the United States in 1960 and has not left the country since.

The patient had six donors, five of whom had no history of malaria, and had negative serologic test results for all four malaria species. Both the patient and his wife had blood smears positive for *P. malariae*. The patient's wife had positive serologic test results for *P. vivax* and *P. ovale* (1:64), for *P. falciparum* (1:258), and for *P. malariae* (1:1024).

**Frances Taylor, M.D., M.P.H.**
Director of Communicable Disease Control, City and County of San Francisco, Department of Public Health, Bureau of Epidemiology, Disease Control, and AIDS, San Francisco, California, USA

### Reference

1. Zucker J. Changing patterns of autochthonous malaria transmission in the United States: A review of current outbreaks. Emerging Infectious Diseases 1996: 2:37-43.

**Reply to F. Taylor:** Dr. Taylor's letter calls attention to the small but important number of induced malaria cases that occur in the United States. From 1957 to 1994, 101 such cases were reported to the Centers for Disease Control and Prevention (CDC); these (including the 1990 case described by Dr. Taylor [1]) are reviewed annually and reported by CDC (2). The occasional occurrence of induced malaria further emphasizes the importance of including malaria in the differential diagnosis of fevers of unknown origin, even in patients who have not traveled to countries where malaria is endemic. Preventing induced malaria requires screening potential blood, tissue, and organ donors and deferring those with a history of malaria or travel to malarious areas. Furthermore, timely surveillance must be maintained to detect induced cases promptly, identify infected blood donors, and prevent additional cases.

The case described by Dr. Taylor was not included in "Changing patterns of autochthonous malaria transmission in the United States: a review of recent outbreaks" (3) because it was a case of induced rather than autochthonous malaria. Each reported malaria case is classified according to standardized terminology (4). *Imported* malaria (which accounts for most cases in this country) is acquired outside the United States and its territories. Malaria acquired within the United States is rare and occurs by one of three mechanisms: *Autochthonous* malaria is acquired through the bite of an infective mosquito. *Congenital* malaria is acquired when a child is infected in utero. *Induced* malaria is transmitted by mechanical means such as transfusion of blood or blood products, organ transplant, deliberate infection for malariotherapy, or contaminated needles or injection equipment. Congenital and induced cases were not included in this review.

When an investigation fails to identify the source of transmission and a case cannot be epidemiologically linked to another case of malaria, the case is classified as *cryptic*. Most cryptic cases are believed to be autochthonous, and there is often evidence to suggest mosquito-borne transmission, even when the source of infection remains unidentified. For this reason, most cryptic cases were included in this review of autochthonous malaria. The two exceptions noted in the article were excluded because both patients had recent histories of blood transfusion, suggesting that their infections were induced.

**Jane R. Zucker, M.D., M.Sc., and**
**S. Patrick Kachur, M.D., M.P.H.**
Centers for Disease Control and Prevention, Atlanta, Georgia, USA

### References

1. Zucker JR, Barber AM, Paxton LA, Schultz LJ, Lobel HO, Roberts JM, et al. Malaria Surveillance—United States, 1992. In: CDC Surveillance Summaries, October 20, 1995. MMWR 1995:44(SS-5):1-17.
2. Centers for Disease Control. Malaria Surveillance Annual Summary, 1990. Atlanta: Centers for Disease Control, 1991.
3. Zucker JR. Changing patterns of autochthonous malaria transmission in the United States: a review of recent outbreaks. Emerging Infectious Diseases 1996;2:37-43.
4. World Health Organization. Terminology of malaria and malaria eradication. Geneva: World Health Organization, 1963:32.

